# Progressive neuropathy in patients with lepromatous leprosy after multidrug therapy

**DOI:** 10.1590/0074-02760220150

**Published:** 2023-01-16

**Authors:** Patricia Sola Penna, Izabela Jardim Rodrigues Pitta, Robson Teixeira Vital, Mariana Andrea Vilas Boas Hacker, Ana Maria Salles, Roberta Olmo Pinheiro, Sergio Luiz Gomes Antunes, Euzenir Nunes Sarno, Márcia Rodrigues Jardim

**Affiliations:** 1 Universidade Federal do Estado do Rio de Janeiro, Programa de Pós-Graduação em Neurologia, Rio de Janeiro, RJ, Brasil; 2 Fundação Oswaldo Cruz-Fiocruz, Instituto Oswaldo Cruz, Departamento de Hanseníase, Rio de Janeiro, RJ, Brasil; 3 Universidade do Estado do Rio de Janeiro, Rio de Janeiro, RJ, Brasil

**Keywords:** lepromatous leprosy, peripheral neuropathy, pathophysiological mechanisms, long-term outcome

## Abstract

**BACKGROUND:**

The lepromatous pole is a stigmatising prototype for patients with leprosy. Generally, these patients have little or no symptoms of peripheral nerve involvement at the time of their diagnosis. However, signs of advanced peripheral neuropathy would be visible during the initial neurological evaluation and could worsen during and after multidrug therapy (MDT). Disabilities caused by peripheral nerve injuries greatly affect these patients’ lives, and the pathophysiological mechanisms underlying nerve damage remain unclear.

**OBJECTIVES:**

To evaluate the outcome of peripheral neuropathy in patients with lepromatous leprosy (LL) and persistent neuropathic symptoms years after completing MDT.

**METHODS:**

We evaluated the medical records of 14 patients with LL who underwent nerve biopsies due to worsening neuropathy at least four years after MDT.

**FINDINGS:**

Neuropathic pain developed in 64.3% of the patients, and a neurological examination showed that most patients had alterations in the medium- and large-caliber fibers at the beginning of treatment. Neurological symptoms and signs deteriorated despite complete MDT and prednisone or thalidomide use for years. Nerve conduction studies showed that sensory nerves were the most affected.

**MAIN CONCLUSIONS:**

Patients with LL can develop progressive peripheral neuropathy, which continues to develop even when they are on long-term anti-inflammatory and immunosuppressive therapy.

Leprosy is a classic example of an infectious neurodegenerative disease of the peripheral nervous system and is one of the leading causes of non-traumatic neuropathy in the developing world.^1^

In patients with lepromatous leprosy (LL), neuropathy usually progresses silently, with widespread involvement of the skin and nerves.^1^ ^,^ ^2^ Some authors have reported that in terms of nerve conduction studies (NCSs), patients with LL worsened overall, and the abnormalities persisted despite an improvement in skin lesions following multidrug therapy (MDT), even in patients without evident neuritis^3^ ^,^ ^4^ ^,^ ^5^ or in those who were treated with corticosteroids.^5^

Despite advances in our understanding of the mechanisms underlying leprosy neuropathy, many questions related to its pathophysiology remain unanswered. This study aims to evaluate neuropathy in a group of LL patients with neurological dysfunction who had nerve biopsies because of worsened symptoms years after completing MDT.

## SUBJECTS AND METHODS

This was a retrospective and descriptive observational study performed by assessing the medical records of the patients who were diagnosed with LL, whose condition worsened at least four years after completing MDT, and who had a nerve biopsy because of the clinical worsening of their symptoms even though they were using corticosteroids or thalidomide.

Patients with comorbidities like diabetes mellitus, vasculitis, hypothyroidism, rheumatological diseases, and human immunodeficiency virus infection that could cause peripheral neuropathy were excluded from this study.

The research was conducted in compliance with the international compilation of human research standards, which was previously approved by the ethics committee of the Oswaldo Cruz Foundation (approval number: 3.152.162). All the patients provided written informed consent.

Patients underwent clinical examinations for the diagnosis of leprosy according to the protocol of the Leprosy Outpatient Unit of the Oswaldo Cruz Institute,^6^ and patients were diagnosed with the lepromatous form of leprosy. They received a fixed-dose multibacillary MDT in accordance with the World Health Organization (WHO) guidelines,^7^ ^,^ ^8^ which were in effect at the time of treatment.

Neurological examinations and NCSs were performed according to the protocol of the Leprosy Outpatient Unit of the Oswaldo Cruz Institute, published elsewhere.^5^ ^,^ ^9^ The disability grade was recorded in accordance with the standard WHO grading criteria.^10^

To evaluate the extent of nerve involvement, neuropathy was classified according to the number of impaired nerves and the distribution of impairment in the NCS. Polyneuropathy is defined as the presence of diffuse, symmetrical peripheral nerve lesions. Furthermore, patients were diagnosed with mononeuropathy when a single nerve was affected and with multiple mononeuropathy when two or more nerves were involved.^11^

Based on the results of compound muscle and sensory nerve action potentials, the categories of nerve segment lesion pathophysiology were defined by combining the NCS parameters. In short, an axonal lesion was defined as either an isolated reduction in amplitude equal to or greater than 30% of the reference values or an amplitude reduction of less than 30% combined with a 60-75% reduction in the conduction velocity of the reference values. Demyelination was verified as a 20% or higher increase in latency, a greater than 35% reduction in conduction velocity, or a combined reduction in amplitude of up to 20% together with a 15-20% increased latency. Demyelinating lesions with axonal degeneration were defined as the presence of axonal and demyelinating lesions within the same nerve. When the action potentials could not be recorded, it was considered that a lesion had “no conduction”.

The sensory nerve was biopsied based on the clinical evaluation of the electrophysiological findings. The following nerves were evaluated: the dorsal cutaneous branch of the ulnar nerve on the dorsum of the hand (n = 7), the sural nerve at the ankle level (n = 6), or the superficial peroneal nerve at the distal third of the leg (n = 1). Nerve samples were analysed according to standard methods.^12^

*Statistical analysis* - Contingency tables were constructed, and data were analysed using the McNemar test to compare the neurological evaluation at the beginning of MDT with that at the time of the biopsy. The means and standard deviations were calculated for continuous variables (age and baseline), and the Mann-Whitney test was used. For comparative analysis of the NCS, a nonparametric Wilcoxon test was used. For clinical electrophysiological comparisons, Fisher’s and chi-square tests were used. A significance level of 5% was considered to be statistically significant.

*Ethics statements* - The research was conducted in compliance with the international compilation of human research standards, which was previously approved by the Ethics Committee of the Oswaldo Cruz Foundation (approval number: 3.152.162). All the patients provided written informed consent.

## RESULTS

*LL patients might show evidence of damage in medium- and large-caliber fibers at diagnosis that is independent of neurological clinical signs* - Table I describes the demographic and clinical characteristics of the patients recruited for this study at the beginning of MDT and at the time of the biopsy. The time between the beginning of MDT and the biopsy ranged from 4 to 16 years (mean age, 8.14 years). Ten patients (71.4%) were male, with ages ranging from 19 to 46 years (mean age, 32.38 years) at the beginning of treatment and from 31 to 61 years (mean age, 40.9 years) at the time of the biopsy. According to the WHO grading system, 46.1% of patients were classified as grade 0 (n = 6). Most patients were classified as grade 1 (46.1%) at the time of the nerve biopsy. According to the Mann-Whitney test (p = 0.003), the average bacilloscopy index at the beginning of treatment was 3.95 (2.16-4.83), and it significantly decreased to an average of 1.0 (0-2.85) at the time of the biopsy.

Symptoms at the beginning of treatment were compared with those at the time of the biopsy using the McNemar test, and only pain showed a significant difference (p = 0.006). Paresthesia and numbness were not significantly different between the two groups (p = 0.102 and p = 0.371, respectively). Regarding the signs of peripheral nerve involvement, we were only able to evaluate thickening using the McNemar test, which showed no significant difference (p = 0.317). Neurological examination showed that most patients already had evidence of damage to medium- and large-caliber nerve fibers at the beginning of treatment (64.3%), which increased to 71.4% of patients at the time of the biopsy (Table I).

**TABLE I:** Demographic and clinical characteristics of recruited patients at the beginning of multidrug therapy (MDT)

Demographic characteristics	At the beginning of MDT	At the time of the biopsy
Age, mean (min-max) in years	32.38 (19-46)	40.9 (31-61)
Gender		
Male	10 (71.4%)	10 (71.4%)
Female	4 (28.6%)	4 (28.6%)
Disability grade		
0	6 (46.1%)	5 (38.5%)
1	4 (30.8%)	6 (46.1%)
2	3 (23.1%)	2 (15.4%)
Bacilloscopy index mean (min-max)	3.95 (2.16-4.83)	1.00 (0-2.25)
Clinical characteristics		
Symptoms (related to peripheral nerves)		
No symptoms	4 (28.6%)	0 (0%)
Pain	1 (7.1%)	9 (64.3%)
Paresthesia	6 (42.9%)	9 (64.3%)
Numbness	4 (28.6%)	1 (7.1%)
Signs (related to peripheral nerves)		
No signs	1 (7.1%)	0 (0%)
Painful and/or thermal impairment	2 (14.3%)	1 (7.7%)
Tactile impairment	6 (42.9%)	5 (35.7%)
Motor	3 (21.4%)	5 (35.7%)
Missing info	2 (14.3%)	3 (23.1%)
Thickening		
Yes	9 (64.3%)	8 (57.1%)
No	4 (28.6%)	4 (28.6%)
Missing info	1 (7.1%)	2 (14.3%)

Time between beginning MDT and worsening of nerve damage in years (min-max): 8.14 (4-16)

**Fig. 1:**
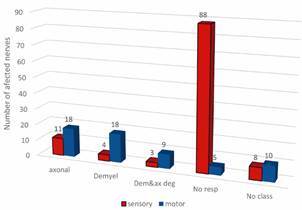
number of affected nerves according to the type of alterations in the nerve conduction studies (NCSs). NCS was performed on 14 patients, and the number of affected nerves was determined based on nerve type (sensory or motor) and type of neural damage. demyel: demyelination; dem & ax deg: demyelinating with secondary axonal degeneration; no resp: no response; no class: no classification.

*NCS shows extensive sensory nerve damage in LL patients since the beginning of MDT* - LL patients underwent NCS at the time of the biopsy (Fig. 1). Sensory NCS was the most affected. Among the evaluated sensory nerves, 77.2% (88 of 114) showed no response, whereas 8.5% (11 of 114) of the nerves with conduction showed axonal lesions. In motor NCS, both axonal and demyelinating changes were present in 30% (18 of 60) of the nerves assessed. In addition, 15% (9 of 60) were demyelinated nerves with secondary axonal degeneration, and 8.3% (5 of 60) showed no response (Fig. 1).

**Fig. 2:**
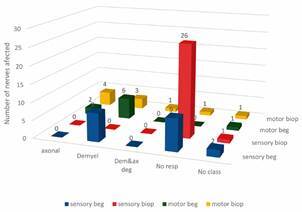
comparison between nerve conduction studies (NCSs) at the beginning of multidrug therapy (MDT) and at the time of the biopsy in four patients. Four patients who presented with NCS at the beginning of treatment were evaluated according to the number of fibers that presented sensory or motor alterations. Each line represents the number of damaged nerves and the type of damage at each time of evaluation. beg: beginning of treatment; biop: time of the biopsy; demyel: demyelination; dem & ax deg: demyelinating with secondary axonal degeneration; no resp: no response; no class: no classification.

Of the recruited patients, only four (all male) underwent NCS at the beginning of treatment, allowing us to evaluate changes in NCS between time points, as shown in Fig. 2. We observed deterioration of NCS findings, with no response in almost all evaluated sensory nerves at the time of the biopsy in comparison with the beginning of MDT. In the motor NCS, there was a reduction in the number of nerves presenting with demyelination. But at the time of the biopsy, there was an increased number of nerves that showed demyelination with secondary axonal degeneration patterns and no response, which showed a worsening of the motor alteration pattern of the peripheral nerve system (Fig. 2).

**Fig. 3:**
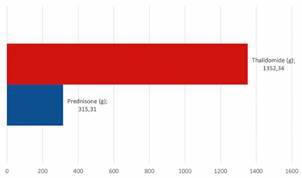
total dosage of medication taken over the years.

*Patients with LL present with worsened neurological symptoms despite the use of prednisone or thalidomide* - In the period between the beginning of the treatment and the nerve biopsy, patients were followed up, and leprosy treatment and reactions were evaluated. During this time, the patients received treatment for neuritis, which worsened despite treatment. They had received a total dosage of 315.31 grams, with an average of 24.25 grams of prednisone (2.32 to 44.03 grams), and a total dosage of 1352.34 grams, with an average of 104.02 grams of thalidomide (0 to 297.4 grams) (Fig. 3). A deterioration of electrophysiological findings was also observed, so a nerve biopsy was recommended for a better evaluation of the condition.

Table II shows the main nerve biopsy findings in the 14 patients. Histopathological analysis showed inflammatory infiltrates in samples from LL patients, 13 (92.9%) of which were positive for acid-fast bacilli. Eleven patients (78.57 %) went through a new cycle of MDT because their symptoms exhibited clinical and neurophysiological worsening and were unresponsive to corticosteroids.

**TABLE II:** Nerve biopsy results

Patient	Nerve	Infl infilt	Granul	Fibrosis	BAAR	Large FR	Small FR	Axon D	Axon R	Demyel
1	Left sural	Yes	1	Yes	2+	NI	NI	NI	NI	NI
2	Right sural	Yes	1	Yes	2+	3	2	0	2	2
3	Right sural	Yes	1	No	Yes	NI	NI	NI	NI	NI
4	Right ulnar	Yes	0	Yes	3+	4	4	0	0	0
5	Right ulnar	Yes	0	Yes	No	3	4	1	2	#
6	Right ulnar	Yes	0	Yes	4+	2	3	0	0	#
7	Left sural	Yes	0	Yes	Yes	4	3	0	1	0
8	Right ulnar	Yes	2	Yes	Yes	2	2	0	2	0
9	Right sural	Yes	0	Yes	2+	4	4	0	0	0
10	Right ulnar	Yes	0	Yes	2+	2	2	1	1	0
11	Right surak	Yes	0	Yes	Yes	NI	NI	NI	NI	NI
12	Left ulnar	Yes	0	Yes	Yes	4	4	0	0	0
13	Left per	Yes	0	Yes	Yes	4	3	0	0	0
14	Right ulnar	Yes	0	Yes	2+	2	3	0	2	0

Per: superficial peroneal; Infl infilt: inflammatory infiltrate; Granul: granuloma; BAAR: acid fast bacilli; FR: fiber reduction; D: degeneration; R: regeneration; Demyel: demyelination; NI: no information.

## DISCUSSION

The early diagnosis of leprosy and adequate therapeutic coverage that reaches all diagnosed individuals are priorities of a leprosy control program, and they are essential for the interruption of transmission and the reduction of the physical and social consequences of the disease.^13^ However, our data showed that this group of patients evolved with neurological worsening despite permanent neurological surveillance.

This group of patients exhibited extensive nerve damage in the absence of symptoms after the diagnosis of the disease. Rambukkana et al.^14^ described the occurrence of early and slowly progressing neurological abnormalities in leprosy even before dermatological lesions occurred. Vital et al.^5^ reported that nerve damage in patients with multibacillary leprosy may occur without symptoms at the onset of the disease.

LL patients may evolve with more extensive neurological involvement, usually without pain, and a slowly progressing evolution. *Mycobacterium leprae* can affect nerves even without activating the inflammatory process, either early in the disease or later on, when the *bacillus* promotes Schwann cell (SC) parasitism and alters nerve function through mechanisms that are yet to be clarified. “Silent neuritis,” or “silent neuropathy” is a condition of sensory or sensory-motor nerve dysfunction that occurs without the pain associated with acute neuritis and evolves indolently, often unnoticed by the patient.^15^ ^,^ ^16^ Medeiros et al.^17^ described a profound metabolic modulation displayed by SC during *M. leprae* infection and hypothesised that lactate reduction in SCs could be an explanation for a new mechanism of demyelination and neuronal death in leprosy neuropathy.^17^

The findings of our study revealed that pain associated with acute neuritis was an unusual symptom at the onset of treatment and that there was a significant increase in chronic spontaneous pain characterised by neuropathic pain. We observed the progression of clinical and electrophysiological changes in the peripheral nerve, with a predominantly sensory onset and progressive compromise in the number of increasingly larger fibers, as occurs in degenerative diseases of the peripheral nervous system, as described by Ooi and Srinivasan.^1^ Rambukkana et al.^18^ proposed that *M. leprae* propagates a nonmyelinating phenotype by inducing demyelination and nerve injury in myelinated SCs in the early phase of infection, thereby possibly explaining the sensitive predominance of peripheral neuropathy.^18^

In our series, nerve damage responded poorly to long treatments with high doses of corticosteroids and/or thalidomide. Rosemberg et al.^19^ found a poor response to corticosteroids in all borderline patients in their study, in which the response was incomplete.^19^ Hulmani et al.^20^ hypothesised that late worsening of symptoms in LL patients may occur due to the persistence of the *bacillus* or reinfection and suggested that patients maintain treatment with MDT until the smear becomes negative. The results of Gondim et al.^21^ demonstrated an increase in cerebrospinal fluid protein levels, suggesting immune-mediated neuropathy in these patients.

We concluded that LL patients can have progressive degenerative peripheral neuropathy from the beginning of the disease, and nerve damage continues to worsen despite treatment with high doses of corticosteroids for years.

Since this was a retrospective study, it has limitations, including the small number of patients and the lack of all the data from the beginning of treatment, such as the NCS. Further studies are needed to better understand the pathophysiology of neuropathy. It is necessary to assess whether axonal degeneration secondary to metabolic alterations in the nerve fiber after SC parasitism may be part of the pathophysiology of this neuropathy, in addition to the known inflammatory response.

We are developing a prospective research project that will measure peripheral neuropathy from the beginning of treatment and throughout the follow-up process at the institution, which would help to assess this outcome better in the future.
